# Exploring Adult Orthodontic Trends on Instagram: A Cross-Sectional Pilot Study of Content, Engagement, and Perceptions

**DOI:** 10.1155/ijod/3508818

**Published:** 2025-11-20

**Authors:** Ramya Vijeta Jathanna, Vinod Rakesh Jathanna, Rithesh Bangera

**Affiliations:** ^1^Department of Orthodontics and Dentofacial Orthopedics, Manipal College of Dental Sciences, Manipal Academy of Higher Education, Manipal, India; ^2^Department of Conservative Dentistry and Endodontics, Manipal College of Dental Science Mangalore, Manipal Academy of Higher Education, Manipal, India; ^3^Department of Orthodontics and Dentofacial Orthopeadics, KVG Dental College and Hospital, Kurunjibhag, Sullia 574327, Karnataka, India

**Keywords:** adult, digital health communication, informal learning, marketing, orthodontics, social media

## Abstract

**Aim or Purpose:**

This study aimed to evaluate the account credentials and content of the top-performing Instagram posts shared publicly about adult orthodontics.

**Materials and Methods:**

A new Instagram account was created and screened manually for this cross-sectional web-based study on March 13, 2024 by using five adult-orthodontics related hashtags. Data was analyzed for post format, content, credentials, role, and engagement level ratio. Unrelated and duplicate posts were excluded from the study. Descriptive statistics were calculated, and comparisons were performed by using the Pearson's Chi-square test and Fisher's exact test (*p*=0.05). This study was exempted from approval by the Institutional Research Committee (IRC) due to the use of publicly available data.

**Results:**

A total of 191,714 posts mentioned the selected hashtags of which top 50 posts were screened in this study. Most of the posts were posted by orthodontists (48%) followed by patients (44%) and other specialists, general dentists, dental lab, and company combined (8%). Most of the posts were in the form of pictures (82%), self-promotional (76%), and used for marketing purposes by orthodontists (89.5%). The most liked videos (7615 likes) were posted by a patient sharing their experience and had an engagement rate of 4.18%.

**Conclusion:**

By leveraging publicly available data, this research provides a foundational understanding of how Instagram serves as a platform for information dissemination and marketing within the field of adult orthodontics. These findings contribute to the broader understanding of digital communication dynamics and underscore the potential of social media platforms for enhancing patient education and professional networking in orthodontic care.

**Clinical Implications:**

This study explored the impact of social media on adult orthodontics by analyzing Instagram posts related to orthodontic treatments, highlighting the diversity of both authors and content. Strengthening the presence of dental professionals on social media platforms could significantly boost the quality and reach of educational content about adult orthodontics, empowering patients with better information and raising awareness about available treatment options.

## 1. Introduction

The advent of social media has profoundly impacted various sectors, including healthcare, by transforming how information is disseminated and consumed [1, 2]. Social media's impact on orthodontics is multifaceted, offering both opportunities and challenges. While it enhances patient engagement and awareness, it also raises concerns about misinformation and unsafe practices. As the landscape continues to evolve, it is crucial for orthodontic professionals to navigate these platforms wisely to ensure safe practices while maximizing the benefits of increased visibility and community support. Instagram, a platform primarily known for its visual content, has become an influential medium for health communication and marketing. Within the field of orthodontics, particularly adult orthodontics, Instagram serves as a vital tool for practitioners to engage with current and prospective patients, share treatment outcomes, and promote their services [3].

The growing trend of adults seeking orthodontic treatment reflects a shift in societal attitudes towards dental health and esthetics [4]. Advances in orthodontic technology, such as clear aligners and less conspicuous braces, have made orthodontic treatments more appealing to adults [5, 6]. Consequently, there is a rising demand for orthodontic care among adults, which orthodontists are increasingly addressing through social media platforms, like Instagram.

Previous research has highlighted the role of social media in healthcare for marketing, patient education, and professional networking [7, 8]. Orthodontists use Instagram to showcase their expertise, share patient testimonials, and provide educational content about orthodontic procedures [9, 10]. This platform allows them to reach a broad audience, including potential patients, who are seeking information and reassurance about orthodontic treatments. Patients, on the other hand, use Instagram to document their orthodontic journeys, share experiences, and connect with others undergoing similar treatments. The engagement and interaction between patients and professionals on Instagram create a dynamic community that enhances the dissemination of information and fosters a sense of support among users [11, 12].

While social media offers benefits, such as professional networking, education, and patient engagement, it also poses risks, including confidentiality breaches and blurred professional boundaries, which are important considerations for orthodontists using Instagram [13].

Despite the growing use of Instagram in adult orthodontics, there is limited research on the specific trends and types of content that drive engagement on this platform. Understanding these trends is crucial for optimizing social media strategies to enhance patient education and engagement effectively. This study aims to evaluate the account credentials and content of the top-performing Instagram posts related to adult orthodontics. By analyzing post formats, content, contributor credentials, and engagement levels, this research provides insights into the digital communication dynamics within the field of adult orthodontics.

## 2. Materials and Methods

**Figure 1:**
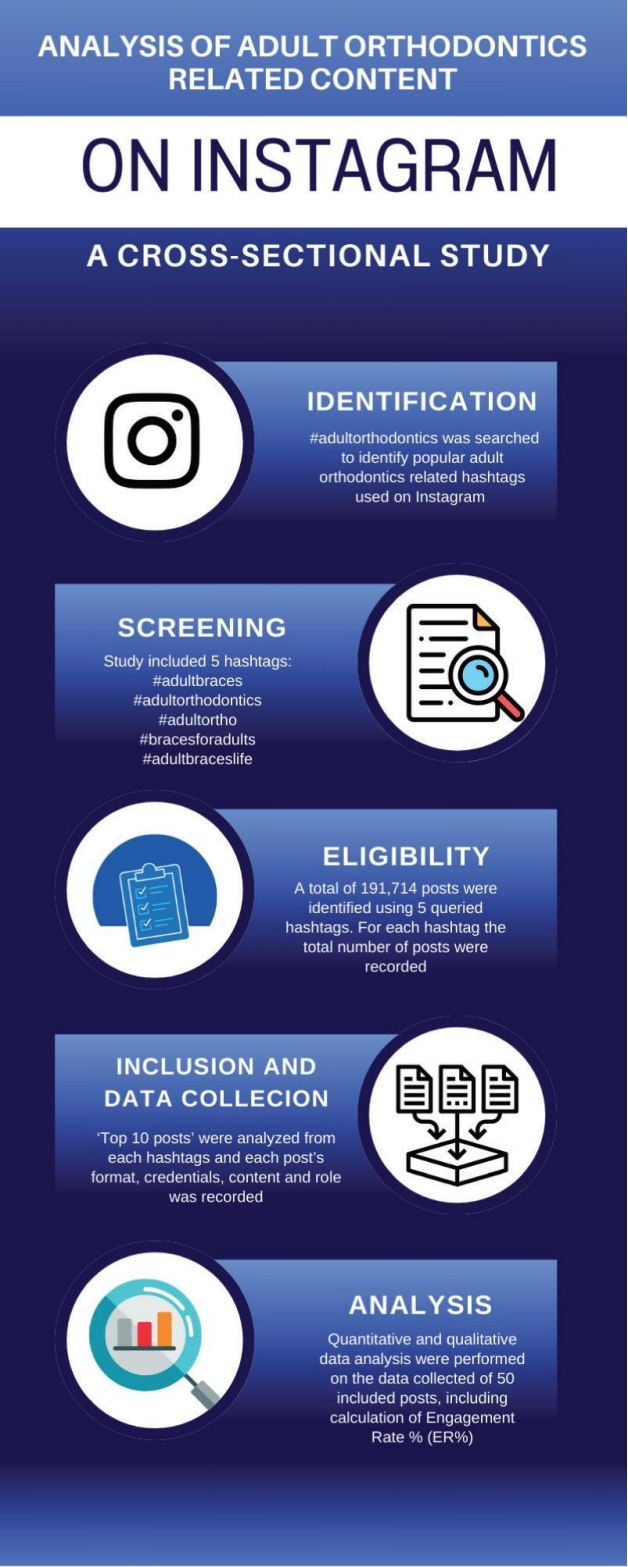
Study flowchart.

This cross-sectional web-based study was designed to evaluate the credentials and content of the top-performing Instagram posts related to adult orthodontics. The study aimed to provide a comprehensive analysis of how adult orthodontic content is shared and engaged with on Instagram. The selection of hashtag, search methods, and data analysis were adapted from previous studies [14, 15]. A new Instagram account was created specifically for the purposes of this study. This account was used to systematically search and screen posts related to adult orthodontics (Figure 1). The systematic search and screening process involved manually retrieving posts displayed under Instagram's “Top posts” category for each selected hashtag on the date of search (March 13, 2024). Posts were screened for relevance by two investigators independently, and disagreements were resolved by discussion. Inclusion criteria required that posts be publicly accessible, directly related to adult orthodontic treatment, and associated with at least one of the selected hashtags. Duplicate and unrelated posts (e.g., posts unrelated to orthodontics or tagged incorrectly) were excluded. Five hashtags relevant to adult orthodontics were selected because they represent the most used and directly relevant search terms for adult orthodontics, based on both preliminary exploration and prior literature examining orthodontic-related content on Instagram. The complete lists of the included hashtags and the total number of posts associated with each are shown in Table 1.

On March 13, 2024, the Instagram account was used to manually search for posts tagged with the selected hashtags (#adultbraces, #adultorthodontics, #adultortho, #bracesforadults, and #adultbraceslife). Five hashtags relevant to adult orthodontics were selected to capture a convenience sample of highly visible posts. The selection was based on Instagram's “Top posts” algorithm, which prioritizes posts with higher engagement (likes and comments). While this approach ensured the inclusion of the most widely viewed content, it does not necessarily represent all posts on Instagram related to adult orthodontics.

The initial search yielded 191,714 posts. Of these, the top 50 posts were selected for detailed analysis. In this study, the choice of 50 posts was made for reasons of feasibility, ensuring that each post could be analyzed in sufficient qualitative and quantitative detail. Moreover, focusing on Instagram's “Top posts” ensured the inclusion of the most widely viewed and engaging content, which is particularly relevant for exploring visibility and patient engagement trends. However, this approach represents only a small proportion of all available posts, and therefore, must be interpreted as exploratory rather than representative. Posts that were unrelated to adult orthodontics or were duplicates of other posts were excluded from the study. However, all 50 of the initially identified posts met the inclusion criteria, and thus, the final sample size for analysis remained 50 posts. However, reliance on Instagram's proprietary “Top posts” algorithm may introduce selection bias, since factors, such as posting time, prior account visibility, paid promotion, or influencer status can influence algorithm-driven rankings.

Each selected post was analyzed for the following parameters: post format (picture or video), content (educational and self-promotional), credentials (orthodontist and patient), role of the person posting (orthodontist, patient, and other dental professionals) and engagement level ratio: metrics, such as likes, comments, and overall engagement rate. Self-promotion was defined as clinician-centered content (e.g., before-and-after photographs, clinic branding, and highlighting qualifications), whereas marketing was defined as campaign-centered content (e.g., advertisements, discounts, and sponsored campaigns). Engagement was defined as “likes” or comments and was calculated for each post separately using Excel (Microsoft Corporation) and the following formula: number of interactions per post/number of followers [16].

### 2.1. Statistical Analysis

Descriptive statistics were calculated to summarize the data. Comparisons between different types of posts and engagement metrics were performed using Pearson's Chi-square test and Fisher's exact test, with a significance level set at *p*=0.05. For exploratory purposes, categorical variables (e.g., post type, content, and source) were converted into dummy variables (0/1 coding), and Pearson's correlation coefficients were calculated to examine associations. Given the nonparametric nature of the data, this approach was intended only as an approximation, and results are interpreted with caution. Formal group comparisons were based on Chi-square and Fisher's exact tests, which are more robust for categorical data.

### 2.2. Ethical Considerations

This study was reviewed by the Institutional Research Committee (IRC) of the Manipal College of Dental Sciences, Manipal Academy of Higher Education, and was granted exemption from formal ethics approval. The exemption was since only publicly accessible Instagram posts were analyzed, with no interaction with users, no collection of personal or identifiable information, and all analyses performed at an aggregate level.

These conditions are consistent with international guidelines the World Medical Association Declaration of Helsinki [17], and the Association of Internet Researchers (AoIR) Ethical Guidelines 3.0 [18], which collectively recognize that research using publicly available, nonidentifiable online data may qualify for exemption.

## 3. Results

### 3.1. Overview of Post Selection

We focused on adult-specific orthodontic hashtag to target explicitly adult treatment; general tags (e.g., #braces and #orthodontics) frequently aggregate adolescent content. Preliminary scoping indicated adult-tagged posts were more likely to contain adult treatment narratives (e.g., clear aligners and esthetic appliances). This topic-delimited strategy aligns with prior work using specialty- or product-specific hashtags [9, 10, 12, 14].

### 3.2. Contributor Analysis

Many of the posts were created by orthodontists, accounting for 48% (24 out of 50) of the top posts. Patients were the second largest group of contributors, responsible for 44% (22 out of 50) of the posts. The remaining 8% (4 out of 50) of posts were contributed by other specialists, general dentists, dental laboratories, and companies.

### 3.3. Post Format

The analysis revealed that pictures were the predominant format, making up 82% (41 out of 50) of the posts. Visual content, particularly before-and-after images, is highly favored for its ability to effectively showcase treatment results and engage viewers. Videos constituted 18% (9 out of 50) of the posts. Despite being less common than pictures, videos often garnered higher engagement rates, likely due to their dynamic and informative nature.

### 3.4. Content Nature

The content of the posts was primarily self-promotional, with 76% (38 out of 50) of posts serving to promote the services of the posting orthodontists. This includes showcasing successful treatments, patient testimonials, and informational content aimed at attracting potential patients.  Table 2 presents both the frequency distribution and percentage of post types across sources to provide a clearer overview of category representation. While orthodontists contributed nearly half of the posts (48%), patients and nonpatients also generated self-promotional content. Taken together, this accounted for 76% of all posts being self-promotional. Among the self-promotional posts by orthodontists, 89.5% (34 out of 38) were explicitly used for marketing purposes. Educational and patient-centered posts comprised a smaller proportion compared to self-promotional content.

### 3.5. Engagement Metrics

One of the standout findings was related to engagement metrics. The most liked video, posted by a patient sharing their orthodontic experience, received 7615 likes and achieved an engagement rate of 4.18%.

### 3.6. Statistical Insights

Descriptive statistics provided a comprehensive overview of the data. Chi-square analyses did not reveal significant associations between post source, type, or content and the number of followers or likes (all *p*  > 0.05). However, correlation analysis demonstrated significant associations between post format and post content (*r* = –0.468, *p*=0.001), post theme and post content (*r* = 0.376, *p*=0.007), and source and post type (*r* = 0.336, *p*=0.017) (Table 3).

## 4. Discussion

The present study offers a exploratory analysis of adult orthodontic trends on Instagram, revealing significant insights into the types of content shared, the profiles of contributors, and the engagement levels of different post formats. Within the limited scope of this pilot study, our findings describe observable patterns in how adult orthodontics is portrayed and engaged with on Instagram. These results should be interpreted as descriptive trends specific to the analyzed sample, rather than as evidence of broader or universal dynamics. While Instagram clearly serves as a platform for both marketing and patient storytelling, the present dataset represents only a very small, highly visible subset of content, and should be seen as exploratory rather than conclusive. Further research comparing multiple platforms (e.g., Facebook, TikTok, and Twitter/X) with larger datasets would be needed to confirm whether similar patterns hold across social media more broadly.

### 4.1. Professional and Patient Contributions

Orthodontists were the primary contributors, responsible for nearly half (48%) of the top-performing posts. This highlights their active participation in leveraging Instagram for professional purposes. The large proportion of orthodontist-generated posts indicate their active use of Instagram for professional promotion. Similarly, the predominance of self-promotional content underscores the platform's marketing role. Patient-generated posts, particularly videos, achieved high engagement, suggesting that authenticity resonates with audiences. Previous studies have noted the increasing use of social media by healthcare professionals for marketing and patient education [3, 7]. Our study supports these findings, demonstrating that orthodontists predominantly use Instagram to showcase treatment outcomes and promote their services. Patients also play a critical role, contributing 44% of the top posts. This significant patient involvement aligns with trends observed in other medical fields, where patients use social media to share personal health experiences and connect with others [13]. Similar patterns have been documented in other fields of medicine where Instagram is used both as a marketing tool and as a channel for patient narratives. For instance, in ophthalmology, Instagram has been used to disseminate clinical information and patient education, though concerns about content accuracy remain [14]. In plastic surgery, studies have found that promotional and esthetic-driven content dominates, paralleling the high proportion of self-promotional orthodontic posts observed in our study [15]. Public health departments have also leveraged Instagram for community engagement and health promotion, but with varying levels of interaction depending on media format and campaign strategy [19]. These cross-specialty comparisons suggest that the predominance of promotional content and the strong audience response to authentic patient experiences may be common features across multiple health domains, not just orthodontics. The high engagement rates of patient-generated content, particularly videos, suggest that authentic patient stories resonate well with audiences, fostering a sense of community and trust.

### 4.2. Content Format and Engagement

The preference for pictures (82%) over videos (18%) among top posts is consistent with Instagram's visual-centric nature. Pictures, especially before-and-after treatment images, are powerful tools for demonstrating the effectiveness of orthodontic treatments.

This visual appeal is crucial in a platform designed to capture attention quickly [8]. for viewers. While both photos and videos are visual, their engagement potential can differ markedly depending on platform, content purpose, and audience expectations. In general, images significantly outperform text-only posts. For example, tweets with images see upto 213% more retweets and 151% more likes [20]. Image quality also matters: richer or professionally composed visuals tend to generate higher emotional and behavioral engagement. Videos, however, often sustain viewer attention longer and foster deeper engagement [21].

The effectiveness of format, nonetheless, is highly platform dependent. On Instagram, carousel images may outperform standalone videos, though reels are often favored for reach and discovery. Media richness theory further supports that more dynamic, multimodal formats, like video enhance message clarity and engagement [22].

Despite their lower frequency, videos generated higher engagement rates. The most liked video, posted by a patient, achieved 7615 likes and an engagement rate of 4.18%. This suggests that while videos are less common, they can be more impactful. Videos provide dynamic content that can offer more comprehensive insights into the orthodontic experience, making them particularly engaging.

### 4.3. Self-Promotion and Marketing

A significant portion of the posts (76%) were self-promotional, with orthodontists using Instagram primarily as a marketing tool. The preference for picture posts over videos was statistically significant (*χ*^2^; test, *p*  < 0.05), as was the high prevalence of self-promotional content among orthodontist-generated posts. This post exemplifies the high engagement potential of authentic patient-generated content, which resonates strongly with viewers. This highlights the strategic use of Instagram by orthodontists to enhance their professional visibility and attract new clients.

This trend is in line with broader social media marketing strategies observed in various professional fields [8, 19, 20]. The fact that 89.5% of orthodontist posts were explicitly for marketing underscores the strategic use of Instagram to attract and retain patients. In our dataset, educational and patient-centered posts comprised a smaller proportion compared to self-promotional content (Table 2). Although underrepresented, these posts are particularly valuable for fostering patient trust, enhancing awareness, and promoting informed decision-making in orthodontic care. Alalawi et al. [23] reported that social media-based dental education improved patient awareness and positively influenced treatment choices, while Casaló et al. [24] highlighted that informative and value-driven posts on Instagram are associated with stronger user engagement and long-term community building. These findings suggest that orthodontists, who increase the proportion of educational and patient-centered content may not only improve patient literacy but also enhance professional credibility and online rapport. Our results, therefore, underscore the untapped potential of educational posts in adult orthodontics, pointing to a need for more balanced content strategies that move beyond self-promotion.

### 4.4. Implications for Practice

The findings of this study have several implications for orthodontic practice. First, orthodontists should consider balancing promotional content with educational and patient-centered posts to enhance engagement and credibility. The success of patient-generated content suggests that encouraging satisfied patients to share their experiences could be an effective strategy to boost engagement [25]. Second, the high engagement rates associated with video content indicate that orthodontists might benefit from incorporating more video posts into their social media strategies. These could include patient testimonials, treatment explanations, and behind-the-scenes looks at orthodontic practice.

### 4.5. Limitations and Future Research

The cross-sectional design provides a snapshot of trends at a single point in time, which may not capture changes over time. Furthermore, reliance on Instagram's “Top posts” algorithm introduces a potential selection bias, as engagement metrics and visibility are shaped by the platform's proprietary ranking system. This may not accurately represent the broader population of adult orthodontic posts on Instagram. Social media trends are dynamic, with rapid shifts in user behavior, platform algorithms, and content strategies. Longitudinal studies are necessary to assess changes over time and to determine causal relationships between content type and engagement. Additionally, the study focused on the top 50 posts, which, while providing insights into the most engaging content, may not represent the full spectrum of posts related to adult orthodontics. While this sampling strategy provided insights into highly visible content, it represents only a very small fraction of all available posts and should not be interpreted as representative of the broader orthodontic discourse on social media. The reliance on top-performing posts introduces an element of selection bias, as engagement metrics are influenced by Instagram's algorithm, posting times, influencer reach, and paid promotion. Therefore, findings should be interpreted as reflecting trends among the most visible content rather than the entire population of posts.

The classification of posts into categories, such as “self-promotional,” “educational,” and “patient-centred” involved a degree of subjectivity, even though coding was performed independently and with consensus. Future studies could improve reliability by employing multiple coders, inter-rater agreement metrics, and more refined classification schemes.

The analysis focused exclusively on Instagram. While Instagram is one of the most widely used platforms in orthodontics, other platforms, such as TikTok, Facebook, and YouTube have different user demographics, content formats, and engagement patterns. Comparisons across platforms are needed to fully understand the role of social media in orthodontic communication.

Future research could expand the sample size and include longitudinal analyses to observe how trends evolve. Investigating the impact of different types of content on patient decision-making and satisfaction could also provide deeper insights. Furthermore, qualitative studies exploring the motivations and perceptions of both orthodontists and patients regarding their social media use could enrich the understanding of digital communication dynamics in orthodontics.

## 5. Conclusion

This cross-sectional analysis of the top 50 Instagram posts related to adult orthodontics provides preliminary insights into the types of content shared, the sources of posts, and the patterns of engagement observed. Self-promotional content was the most common, while educational and patient-centered posts, though less frequent, demonstrated potential value for enhancing patient engagement and professional credibility. These findings should be interpreted cautiously given the limited dataset and platform-specific focus. Rather than definitive conclusions, the present study offers a starting point for understanding how orthodontics is represented on Instagram and underscores the need for more extensive, multiplatform, and longitudinal research to better capture evolving trends in digital orthodontic communication.

## Figures and Tables

**Table 1:** Adult-orthodontics related hashtag utilization on Instagram.

Hashtag	Number of posts
#adultbraces	155,669
#adultorthodontics	20,049
#adultortho	5951
#bracesforadults	5592
#adultbraceslife	4452

**Table 2:** The cross-tabulation of post source and content type.

Source	Self-promotional (*n*, %)	Educational (*n*, %)	Informational/other (*n*, %)	Total (%)
Orthodontists	28 (56)	12 (24)	8 (20)	48
Patients	9 (18)	2 (4)	11 (22)	44
Non-patients	1 (2)	0 (0)	1 (2)	4
Total	38 (76)	14 (28)	20 (40)	100*⁣*^*∗*^

*⁣* ^*∗*^Percentages are relative to total posts (*N* = 50). Frequency distributions (*n*) are included to illustrate raw counts within each category.

**Table 3:** Summary of statistical analyses of Instagram post characteristics and engagement metrics.

Comparison	Test used	Statistic	*p*-Value	Significant
Source vs. number of followers	Pearson *χ*^2^	92.7 (df = 82)	0.197	No
Post type vs. number of followers	Pearson *χ*^2^	142.7 (df = 123)	0.108	No
Post content vs. number of followers	Pearson *χ*^2^	50.0 (df = 41)	0.158	No
Post format vs. number of likes	Pearson *χ*^2^	31.4 (df = 38)	0.768	No
Post format–post content	Pearson correlation	−0.468	0.001	Yes
Post theme–post content	Pearson correlation	+0.376	0.007	Yes
Source–post type	Pearson correlation	+0.336	0.017	Yes

## Data Availability

The data that support the findings of this study are available from the corresponding author upon reasonable request.
